# Deep Spectral-Spatial Features of Near Infrared Hyperspectral Images for Pixel-Wise Classification of Food Products

**DOI:** 10.3390/s20185322

**Published:** 2020-09-17

**Authors:** Hongyan Zhu, Aoife Gowen, Hailin Feng, Keping Yu, Jun-Li Xu

**Affiliations:** 1 College of Electronic Engineering, Guangxi Normal University, Guilin 541004, China; hyzhu@mailbox.gxnu.edu.cn; 2 UCD School of Biosystems and Food Engineering, University College of Dublin (UCD), Belfield, Dublin 4, Ireland; aoife.gowen@ucd.ie; 3 School of Information Engineering, Zhejiang Agricultural and Forestry University, Hangzhou 310000, China; hlfeng@zafu.edu.cn; 4 Global Information and Telecommunication Institute, Waseda University, Shinjuku, Tokyo 169-8050, Japan; keping.yu@aoni.waseda.jp

**Keywords:** hyperspectral, spatial-spectral features, classification, principal component analysis, convolutional neural network

## Abstract

Hyperspectral imaging (HSI) emerges as a non-destructive and rapid analytical tool for assessing food quality, safety, and authenticity. This work aims to investigate the potential of combining the spectral and spatial features of HSI data with the aid of deep learning approach for the pixel-wise classification of food products. We applied two strategies for extracting spatial-spectral features: (1) directly applying three-dimensional convolution neural network (3-D CNN) model; (2) first performing principal component analysis (PCA) and then developing 2-D CNN model from the first few PCs. These two methods were compared in terms of efficiency and accuracy, exemplified through two case studies, i.e., classification of four sweet products and differentiation between white stripe (“myocommata”) and red muscle (“myotome”) pixels on salmon fillets. Results showed that combining spectral-spatial features significantly enhanced the overall accuracy for sweet dataset, compared to partial least square discriminant analysis (PLSDA) and support vector machine (SVM). Results also demonstrated that spectral pre-processing techniques prior to CNN model development can enhance the classification performance. This work will open the door for more research in the area of practical applications in food industry.

## 1. Introduction

Hyperspectral imaging (HSI) was originally developed in the early 1970 for remote sensing applications [1]. The invention of the first charge-coupled device (CCD) detector played a crucial role in pushing this technology forward. In recent years, the technology has been reported to have applications in many diverse fields such as forensic science [2], pharmaceutical research [3], agriculture [4], and food science [5]. HSI goes beyond traditional imaging techniques by integrating spectral and spatial information from an object [6]. Therefore, the merits of spectroscopy and computer vision are both reflected in hyperspectral imaging. Spectroscopy identifies the analyte of interest based on its spectral signature, and imaging transforms this information into distribution maps for spatial visualization.

A key step in the successful implementation of HSI applications is the development of calibration models. For a classification task on near infrared hyperspectral imaging dataset acquired from benchtop instruments, chemometrics is currently considered as a popular tool that has been used for many years. Partial least squares discriminant analysis (PLSDA) [7] is a supervised classification modelling method that uses the PLS algorithm to predict the belonging of a sample to a specific class. PLSDA is increasingly used in hyperspectral data analysis for classification problems due to its capability to deal with multicollinearity problem in near infrared (NIR) spectra, which occurs because of very high intercorrelation between absorbances [8,9]. Nevertheless, only spectral features were used as input for classification models in most cases.

Machine learning (ML) techniques have been introduced for HSI data classification [10], which have been collected in an extensive list of detailed reviews, such as Li, et al. [11,12]. The ML field has experienced a significant revolution thanks to the development of new deep learning (DL) models since the early 2000s [13], which is supported by advances in computer technology. These models have gained popularity in the development of HSI classifiers [13,14]. For instance, support vector machine (SVM) was applied to HSI data for strawberry ripeness evaluation achieving classification accuracy over 85%. Convolutional neural network (CNN), being the current state-of-the-art in deep learning [15], first achieved success in the field of image recognition and has become an extremely popular tool for remotely sensed HSI data classification [10]. More importantly, CNN models show flexibility to deal with HSI data by introducing a one-dimensional CNN for processing spectral inputs [16], two-dimensional CNN for single or multiple wavelength images [17], and three-dimensional CNN (3D-CNN) for an intelligent combination of spectral and spatial image data [18,19]. Although CNN models have been successfully implemented for remote sensing applications, they are not often applied to HSI data of food products. Earlier this year, Al-Sarayreh, et al. [20] reported that 3D-CNN model approach applied to HSI data significantly enhanced the overall accuracy of red meat classification.

In this context, the current work aims to investigate the advantages and disadvantages of applying deep learning approaches to near infrared HSI data. The main objective is to compare CNN based modelling strategies against traditional chemometric (i.e., PLSDA) and machine learning (i.e., SVM) methods for the pixel-wise classification tasks of food products. We will apply a hybrid framework which involves first performing principal component analysis (PCA) to highlight the major spectral variation and then building 2-D CNN model from the first few PCs using spatial features. PLSDA, SVM and 3-D CNN are also applied for classifying HSI image data. The performance will be evaluated and compared in terms of efficiency and accuracy, exemplified through two case studies, i.e., classification of four sweet products and differentiation between white stripe (“myocommata”) and red muscle (“myotome”) pixels on salmon fillets.

## 2. Materials and Methods

### 2.1. Sweets Dataset

Spectral images of the sweet samples are acquired in the reflectance mode by employing a laboratory-based pushbroom hyperspectral imaging system. The main components of this system are an imaging spectrograph (Specim N17E, Spectral Imaging Ltd., Oulu, Finland) and an InGaAs camera (InGAs 12-bit SU320MS-1.7RT Sensors Unlimited, Inc., Princeton, NJ, USA). This configuration captures an image of a line across the sample, spanning the 320-pixel width of the sensor, and the spectrograph produces a spectrum for each of these pixels across the other dimension of the array, accounting for a two-dimensional image. The wavelength interval is 7 nm in the spectral range of 943–1643 nm, leading to 101 spectral bands. Direct reflectance spectra are used for subsequent data analysis.

This dataset consists of NIR hyperspectral images of four different sweets with different shapes, colors, and nutritional compositions among classes. Specifically, the details of the selected four products are as follows: raspberry flavor mushroom in pink and white color with a mushroom shape, mint humbugs in brown and golden stripe with an ellipse shape, teeth and lips in pink and white color with a teeth-like appearance, and tub in brown with a cola bottle shape. These sweets, made and purchased from Tesco Ireland Ltd., are labelled as raspberry flavor mushroom (RFM), Mint, Teeth, and Tub, respectively. Sweet samples are chosen because the different spatial information among classes has great potential for improving classification performance.

**Figure 1:**
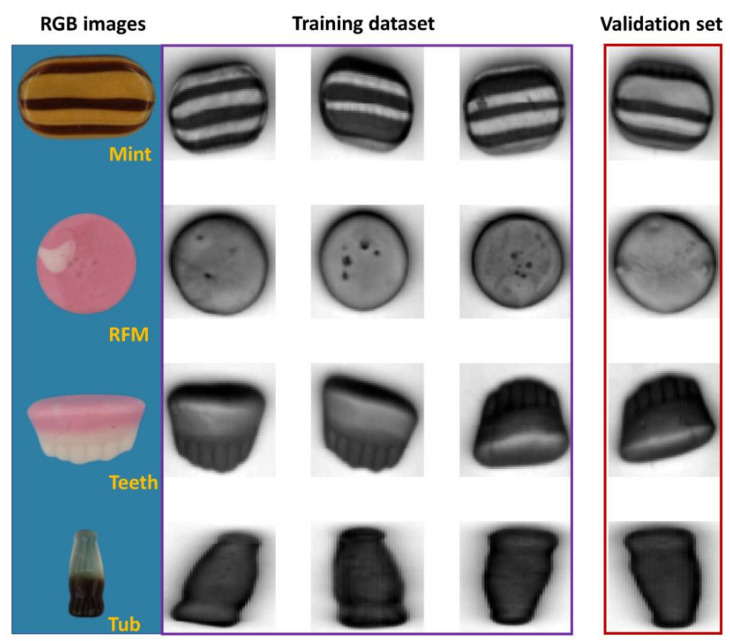
The RGB images of sweet samples and mean images at the spectral domain.

Four hypercubes of each sample type are obtained with the mean image showing in Figure 1, together with the representative red/green/blue (RGB) images captured by a computer vision system as described in Xu and Sun [21]. As seen, the first three samples of each type are selected as the training set for model development, while the remaining one serves as the validation set, leading to 12 hypercubes consisting of 27,807 pixels for training and 4 hypercubes of 8947 pixels for validation purpose. In addition to this, the developed model is tested on two mixed images containing two samples of each material. The mean images of the mixture are shown in Figure S1 of Supplementary Materials.

### 2.2. Salmon Dataset

NIR spectral images of farm-raised Atlantic salmon (*Salmon salar*) fillets are also collected in the reflectance configuration. The core components of the system included: an imaging spectrograph (ImSpector, N17E, Spectral Imaging Ltd., Oulu, Finland) collecting spectral images in a wavelength range of 900–1700 nm, a high performance camera with C-mount lens (Xeva 992, Xenics Infrared Solutions, Leuven, Belgium), two tungsten-halogen illuminating lamps (V-light, Lowel Light Inc., New York, NY, USA), a translation stage operated by a stepper motor (GPL-DZTSA-1000-X, Zolix Instrument Co., Beijing, China), and a computer installed with a data acquisition software (SpectralCube, Spectral Imaging Ltd., Oulu, Finland). Each fillet was individually placed on the moving table and then was scanned line by line at a speed of 2.7 cm/s adjusted to provide the same vertical and horizontal resolution (0.58 mm/pixel).

Salmon is valued as a fat-rich fish with a large proportion of lipids congregated in the white stripe of connective tissue (“myocommata”), segmenting the red-colored muscle (“myotome”) tissue in vertical blocks and presenting a zebra-like appearance [22]. Previous study has demonstrated that the proportion of myocommata in a salmon fillet correlated well with its fat content [23]. In this sense, it is interesting to classify the white stripe from the red muscle because the proportions of the white strip in one fillet might contain some valuable information about the fat content and/or lipid oxidation [24]. The salmon dataset is of interest because there is spatial information on the salmon surface, yet it is unsure if this spatial information could help classification.

**Figure 2:**
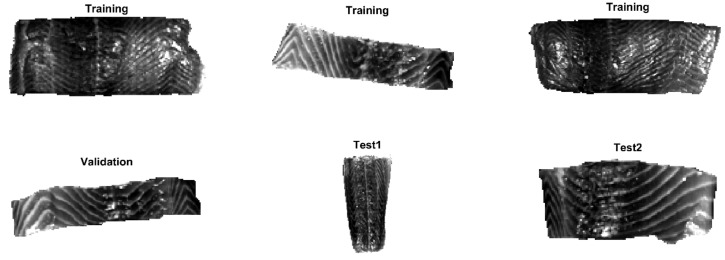
Mean images of salmon fillets at the spectral domain.

Overall, six salmon fillets are used with the mean images shown in Figure 2. The first three samples are included in the training set to develop classifiers, while the fourth sample is used as the validation set and the remaining two images are considered as the test set. These salmon fillets are obtained from three different batches. As seen from Figure 2, they are cut from different positions of fish. It can be discerned that samples are in different sizes and shapes. Some pixels with strong signals are also observed, which poses some challenges for the pixel classification. There could be due to the specular reflection of the illumination source at the salmon surface to produce regions with high-intensity values in the hyperspectral images. These regions act like a mirror and lead to saturation of CCD because of the white stripe or the existence of scales.

### 2.3. Background Removal

All data analysis is carried out using MATLAB (release R2019a, The MathWorks, Inc., Natick, MA, USA) incorporating functions from Deep Learning Toolbox, Statistics, and Machine Learning Toolbox, and additional functions written in-house.

Sweet samples are placed on a white tile for imaging. Background removal is carried out by subtracting a low-reflectance band from a high-reflectance band followed by a simple thresholding. In this context, the reflectance image at 1496 nm is subtracted from that of 957 nm to enhance the contrast between the sample and white tile. Afterwards, a threshold of 0.22 is applied for background removal. A binary mask for background removal is subsequently generated with all background regions set to zero.

Salmon samples are directly placed on the moving table for image acquisition. The background is removed from the salmon flesh image in the same manner. Bands 944 nm and 1450 nm are used followed by a thresholding value of 0.2. However, the selected bands and thresholding values might change depending on the segmentation result of individual hyperspectral image.

### 2.4. Spectral Pre-Processing

In the field of hyperspectral imaging, the most common practice is the adaptation of different pre-processing techniques [25]. Spectral preprocessing algorithms are mathematically used to improve spectral data. It aims to correct undesired effects such as random noise, length variation of light path, and light scattering resulting from variable physical sample properties or instrumental effects. This step is generally performed prior to multivariate modelling so as to reduce, eliminate, or standardize the impact on the spectra and to greatly enhance the robustness of the calibration model [26]. In this work, three spectral pre-processing methods are attempted comparatively, namely, standard normal variate (SNV), first derivative, and external parameter orthogonalization (EPO [27]). SNV is a mathematical transformation method of spectra, which is used to remove slope variation and correct light scattering effects. As one of the normalization methods, SNV is performed by first calculating the standard deviation and then normalizing by this value, thus giving the sample a unit standard deviation. First derivative using Savitzky-Golay (SG) method can reduce additive effects [28]. EPO decomposes a spectrum into two components: a useful component that has a direct relationship with the response variable, and a parasitic component that is from an external influence [29]. By removing the parasitic component through orthogonal transformation of spectra, the calibrated spectral model can be less sensitive to the external influence.

### 2.5. PLSDA and SVM Modelling

Partial least squares discriminant analysis (PLSDA) [30] and SVM are used to build classification models. It is common practice to “unfold” hypercubes such that the three-dimensional information is presented in two dimensions. Unfolding simply refers to rearranging spectra from a hypercube with three dimensions ((1) rows, (2) columns, and (3) wavelengths) to a matrix with two dimensions ((1) rows × columns against (2) wavelengths). Non-background pixels are extracted from each hypercube by unfolding and concatenated to make a two-dimensional matrix (X, i.e., a matrix where the rows represent observations and columns represent spectral features). PLSDA and SVM models are developed from X and Y (i.e., a matrix where the rows represent observations and columns represent the true classes). It is significant to select the proper number of latent variables (LVs). Inclusion of too few or too many LVs may lead to, respectively, under or over-fitting of the data and subsequently lead to poor future model performance [31]. In this work, venetian blinds cross-validation is applied to determine the optimal number of LVs, which is performed by checking the evolution of the accuracy with the number of LVs.

Multiclass support vector machine (SVM) with the error correcting output codes (ECOC) is also implemented for comparison. The SVM is a binary classifier which can be extended by fusing several of its kind into a multiclass classifier [32]. In this work, SVM decisions are fused using the ECOC approach, adopted from the digital communication theory [33].

### 2.6. PCA-CNN Modelling

This method starts with employing PCA on the global dataset to seek for the spectral variance among different sample types. To do this, it is necessary to unfold all image cubes in the training set along the spatial axis and takes all of the pixel spectra from each hypercube (omitting the background) and then concatenates them to make a two-dimensional matrix on which PCA is performed. PCA decomposes the original data matrix into scores and loadings. Each loading is a vector which provides information on the relative importance, or the weighting, of specific wavelengths relative to each other. The first PC describes the largest variance in the dataset and each following PC describes progressively less of the variance. Therefore, instead of using all loading vectors, we can opt to just use some of the earlier loading vectors to represent the original dataset. For samples in validation and test sets, the individual hypercube is first unfolded and then projected along the PC loadings by matrix multiplication producing PC scores matrices which are subsequently re-folded to form score images.

Score images from the first few PCs are used as the input for 2-D CNN model. For a pixel-based classification of hypercube $I\left(x,\;y,\;\lambda \right)$, where x and y are the width and the height of the image and λ denotes the number of spectral bands, it aims at predicting the label of each pixel within the image. Initially, the original hypercube $I\left(x,\;y,\;\lambda \right)$ is reduced to score images with the size of x×y×d where d refers to the number of selected PCs. The next step is to extract a k×k×d patch for each pixel, where k denotes the window size of the patch. In specific, each patch (i.e., the spatial context) is constructed surrounding a pixel, the center point of the patch. For the pixels that reside near the edge of the image, the patch includes some pixels belonging to the sample while the others belonging to the background.

In this work, the structure of the 2-D CNN consists of an input layer, a convolution (Conv) layer, a rectified linear unit (ReLU) layer, a pooling (POOL) layer, a dropout layer, a fully connected (FC) layer, a softmax layer, and an output layer. The convolutional layer convolves the input data by applying sliding convolutional filters and outputs the convolved features [34], that is, the feature maps. Each convolutional kernel outputs a feature map corresponding to a type of extracted features. Traditional convolution moves from left to right and from top to bottom with a step of 1. Strided convolution has a larger and user-defined step size for traversing the input. All feature maps are stitched and merged by the first fully connected layer to summarize all local features. The number of neural nodes in the fully connected layer changes with the convolution kernel size, the sampling kernel size, and the number of feature maps. For a classification task, it is a common practice to place a softmax layer after the last FC layer. The softmax function is used to compute the probability that each input data pattern belongs to a certain class. The kernel size, the number of feature maps, and the spatial size (i.e., the window size of the patch) are critical parameters in CNN model. These parameters were optimized based on a systematic way of tuning one parameter and fixing it followed by the same procedure for others.

### 2.7. Three-Dimensional CNN Modelling

As the image formed by hyperspectral bands may have some correlations, e.g., close spectral bands may account for similar images, it is desirable to take into account spectral correlations. Although the 2-D CNN model enables to use the spatial context, it is applied without consideration of spectral correlations. To address this issue, a 3-D CNN model is proposed to extract high-level spectral-spatial features from the original 3-D hyperspectral images. A patch (k×k×λ) for each pixel is extracted from hypercube and used as the input. The operational details of the 3-D CNN model are quite similar to those of the 2-D CNN model. Different from 2-D CNN, the convolution operator of this model is 3-D, whereas the first two dimensions are applied to capture the spatial context and the third dimension captures the spectral context. In addition to a Conv layer, a BN layer, a ReLU layer, a dropout layer, a FC layer, a softmax layer are included in the designed network structure.

### 2.8. Assessment of Classification Models

Essentially, the performance of a classifier is assessed by the data set classification accuracy index, i.e., % correct classification rate (%CCR). The ground truth for sweet samples is directly obtained by labelling after removing background (see Section 2.3). For salmon samples, a local thresholding strategy is applied on PC2 score images to obtain ground truth. Firstly, individual score image is divided into several sub-images and then an optimal threshold value is manually selected for each sub-image. Confusion matrix, also known as an error matrix, is used to evaluate the quality of the output of the classifier for validation and test sets. The elements in the diagonal are the elements correctly classified, while the elements out of the diagonal are misclassified. We also compute the percentages of all the examples belonging to each class that are correctly and incorrectly classified and show them on the far right of the confusion matrix. These metrics are often called the recall, also known as sensitivity (or true positive rate (TPR)) and false negative rate (FNR), respectively. The row at the bottom of the confusion matrix shows the percentages of all the examples predicted to belong to each class that are correctly and incorrectly classified. These metrics are often known as the precision (or positive predictive value (PPV)) and false discovery rate (FDR), respectively. In detail, they are calculated as below:

(1) $$TPR=\frac{TP}{TP+FN}\;\;\;$$

(2) $$FNR=\frac{FN}{FN+TP}\;\;\;\;$$

(3) $$PPV=\frac{TP}{TP+FP}$$

(4) $$FDR=\frac{FP}{FP+TN}\;\;\;\;$$

In these equations, *TP*, *TN*, *FP* and *FN* respectively refer to true positive, true negative, false positive and false negative. Desirable classification performance is characterized with higher CCR, *TPR*, *PPV*, and lower *FNR* and *FDR*. Apart from these, classification and misclassification maps are also displayed to visualize where are the correctly and incorrectly classified pixels.

## 3. Results

### 3.1. Results of Sweet Dataset

#### 3.1.1. Spectral Pre-Processing

**Figure 3:**
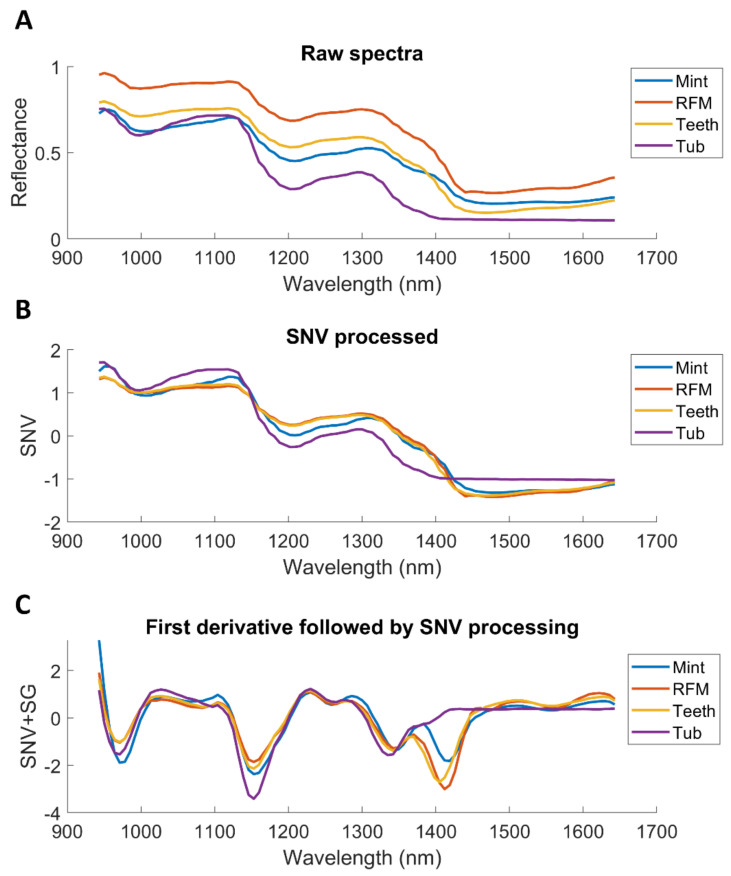
Influence of different spectral pre-processing methods on the mean spectra of one hypercube in the training set. (**A**) Raw spectra; (**B**) Pre-processed spectra with standard normal variate (SNV); (**C**) First derivative spectra (Saviztky-Golay with a window size of 11 and third order polynomial degree) followed by SNV.

Figure 3 shows the spectra averaged from one hypercube in the training set and the outcome of pre-treatments. RFM presents the highest reflectance across the whole spectral region (see Figure 3A), while Tub is the lowest. Discrimination among sweet types is highly possible owning to the observable difference in spectral profiles. SNV pre-processed results are displayed in Figure 3B. The combination of SNV and first derivative (window size of 11 and third order polynomial degree) is also applied, as shown in Figure 3C. It is noticed that the spectral difference among sweet samples is highlighted over 1400–1500 nm, which is related to water band due to hydrogen bonding [35]. 

#### 3.1.2. PLSDA and SVM Modelling

Figure S2 shows the evolution of accuracy (%) with the number of LVs. In general, CCR (%) increases rapidly at the first few LVs and then remains constant, i.e., adding more variables will not improve accuracy. To avoid both underfitting and overfitting, LVs that contributed most to the enhancement of accuracy were selected, as per the outcome shown in Figure S2. The classification model performance of PLSDA and SVM, in terms of CCR (%) calculated on the validation set and prediction images, are shown in Table 1. In general, the accuracy was found to be higher than 99% for validation and test sets, suggesting the classifiers can generalize well on unknown samples. Apparently, pre-treatments enable the enhancement of the model’s performance, as evidenced by the increased accuracy of test sets. It is also noticed that SVM outperformed PLSDA under the condition of using the same spectral pre-treatment.

**Figure 4:**
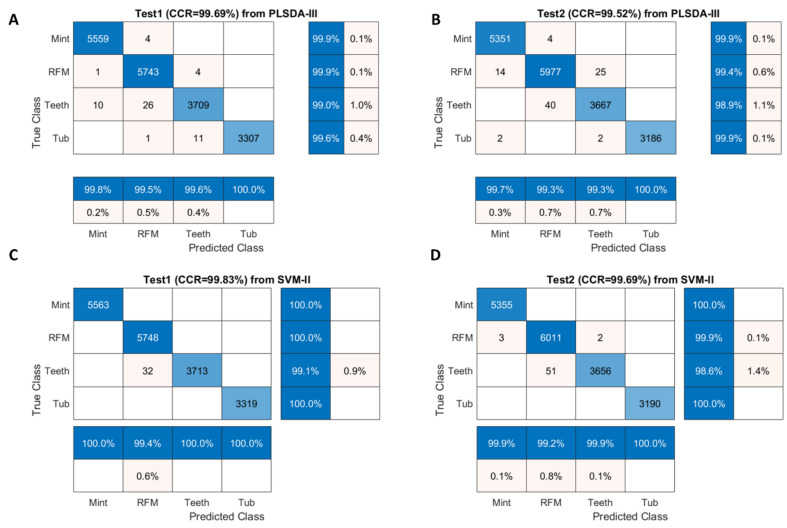
Confusion matrices for Test 1 image (**A**) and Test 2 image (**B**) obtained from PLSDA-III model; for Test 1 image (**C**) and Test 2 image (**D**) obtained from SVM-II model.

Confusion matrices for test sets were obtained for PLSDA-III (pre-processed with SNV combined with first derivative) and SVM-II (pre-processed with SNV) and displayed in Figure 4. As illustrated, classification of Teeth pixels had the lowest TPR (i.e., sensitivity), suggesting that the true Teeth pixels are less likely to be recognized. Indeed, 10 pixels of Teeth were wrongly classified as Mint and 26 pixels as RFM in Test 1 image (Figure 4A), while 40 pixels of Teeth were incorrectly identified as RFM for Test 2 (Figure 4B), in consistent with Figure 3C where Teeth and RFM present close spectral profiles spanning the entire spectral region. The same observation can be found from SVM modelling result, in which 32 Teeth pixels were wrongly classified as RFM for Test 1 image (Figure 4C) and 51 pixels misclassified as RFM for Test 2 image (Figure 4D).

**Figure 5:**
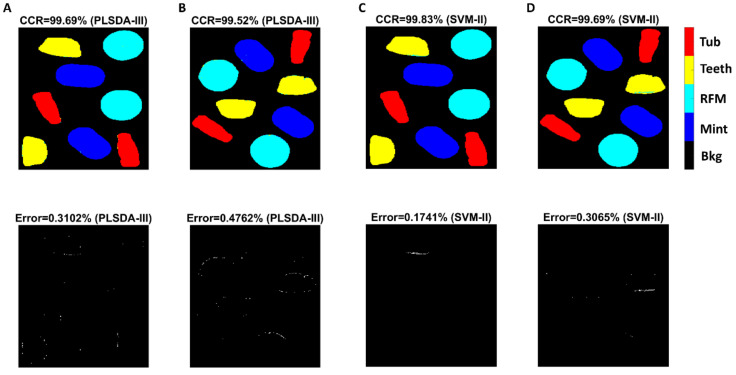
Classification and misclassification maps for Test 1 image (**A**) and Test 2 image (**B**) obtained from PLSDA-III model; for Test 1 image (**C**) and Test 2 image (**D**) obtained from SVM-II model.

In order to produce classification maps, the mixture image was first unfolded with background pixels removed using masking to form a two-dimensional matrix on which the developed classifier could be applied. Finally, the resultant matrix with the predicted class assigned to each pixel needed to be refolded to generate classification maps, as shown in Figure 5. It is observed that most misclassification pixels are distributed along the edge of each object. It is also seen that SVM modelling produced less misclassified pixels.

#### 3.1.3. PCA-CNN Modelling

**Figure 6:**
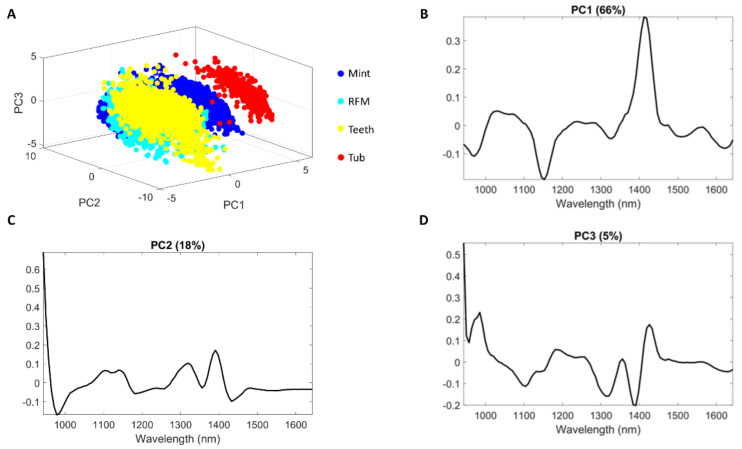
Principal component analysis score plots (**A**) and loading for PC1 (**B**), PC2 (**C**) and PC3 (**D**).

First derivative (Saviztky-Golay with a window size of 11 and third order polynomial degree) followed by SNV pre-treated spectra were used to build PCA, with the first three PCs displayed in Figure 6. The pixels belonging to Tub can be easily separated from Teeth and RFM on PC1. The loading plot indicates that the band around 1410 nm mainly contributed to this separation, in agreement with the spectral profiles of Figure 3C. PC1 score images are shown in Figure S3 where different spatial patterns among classes are clearly perceivable, suggesting the suitability of using 2-D CNN modelling subsequently. Figure S4 showing the PC2 score image demonstrates that Mint pixels have higher values (red color) compared to others (blue color), implying the potential of separating Mint from other classes.

The original hypercube with 101 spectral variables was transformed to score images with the first 10 PCs (explaining 97% of variance) selected, followed by patch extraction with the window size of 17. For the training of 2-D CNN, the learning rate was set to 0.01, and the epoch of training was set to 100, the mini-batch size was set to 1024. Convolution layer was implemented with 20 feature maps (filter size = 5 × 5, stride = [2 2]), while the height and the width of the pool size are set to 2 with a stride of 2.

The accuracy and loss for training and validation sets were plotted against the number of iterations, as shown in Figure S5. The accuracy for training and validation both increase and then remain flat. The loss on the training set decreased rapidly for the first 100 iterations, suggesting that the network was learning fast to classify sweet samples. The loss of the validation set also decreased fast and stayed roughly within the small range as the training loss, implying that this model generalizes reasonably well to unseen data.

PCA-CNN model performances are also illustrated in Table 1. As shown, spectral pre-processing enabled the improvement of the model’s performance, for instance, from the accuracy of 99.00% using raw spectra to accuracy of 100% using SNV pre-treated spectra. Compared to PLSDA and SVM modelling, PCA-CNN-III (pre-processed with first derivative followed by SNV) facilitated better predictive ability, providing 100% accuracy in validation and test sets. In terms of efficiency, PCA-CNN-III required two minutes for processing, which is acceptable compared to SVM and PLSDA.

#### 3.1.4. Three-Dimensional CNN Modelling

The same pre-processing procedures (SNV, SG+SNV) were carried out in order to compare with raw spectra, after which a patch with the size of 7×7×101 was extracted from hypercube and used as the input. The same learning rate, and the number of epoch (see Section 3.1.3) were utilized in 3-D CNN model training. Convolution layer was performed with 3-D convolution operator (10 feature maps, filter size = 3 × 3 × 10, stride = [1 1 1]) with the training progress shown in Figure S6. Compared to PCA-CNN model (Figure S5), similar curve shapes (i.e., accuracy and loss) are noticed. Likewise, accuracy first soars and then remains stable after 400 iterations, while loss declines fast at the beginning and keeps flat, indicating that the model was not under or over-fitted.

The model performance of 3-D CNN is also displayed in Table 1. Again, we can observe that spectral pre-processing techniques greatly enhance classification performance. The accuracy of 100% was obtained from pre-processed spectra for training, validation, and both test sets. However, 3D-CNN-I model built from raw spectra delivered the worst performance with the lowest accuracy (in terms of validation and test sets) compared to other models. This suggests that spectral pre-treatment plays an important role in improving the effectiveness of 3-D CNN model developed from near-infrared HSI dataset.

The advantage of 3-D CNN is that it can exploit the spatial and spectral context simultaneously. In essence, PCA can exploit the spectral features, and then 2-D CNN can exploit the spatial context; therefore, the PCA-CNN method also enables the extraction of joint spectral-spatial information from each hypercube. Sweet samples suggest that PCA-CNN and 3-D CNN models after spectral preprocessing, i.e., PCA-CNN-III, 3D-CNN-II, and 3D-CNN-III from Table 1, delivered the best predictive ability with 100% accuracy for validation and test sets. Nevertheless, 3-D CNN required much longer time for the training process, i.e., 2 min of training 2-D CNN model versus 13 min of training 3-D CNN model using the same computer. Indeed, 3-D CNN model brings complexity into the classifier, increasing the number of parameters that each neural model needs to adjust during the training phase.

### 3.2. Results of Salmon Dataset

#### 3.2.1. Spectral Pre-Processing

**Figure 7:**
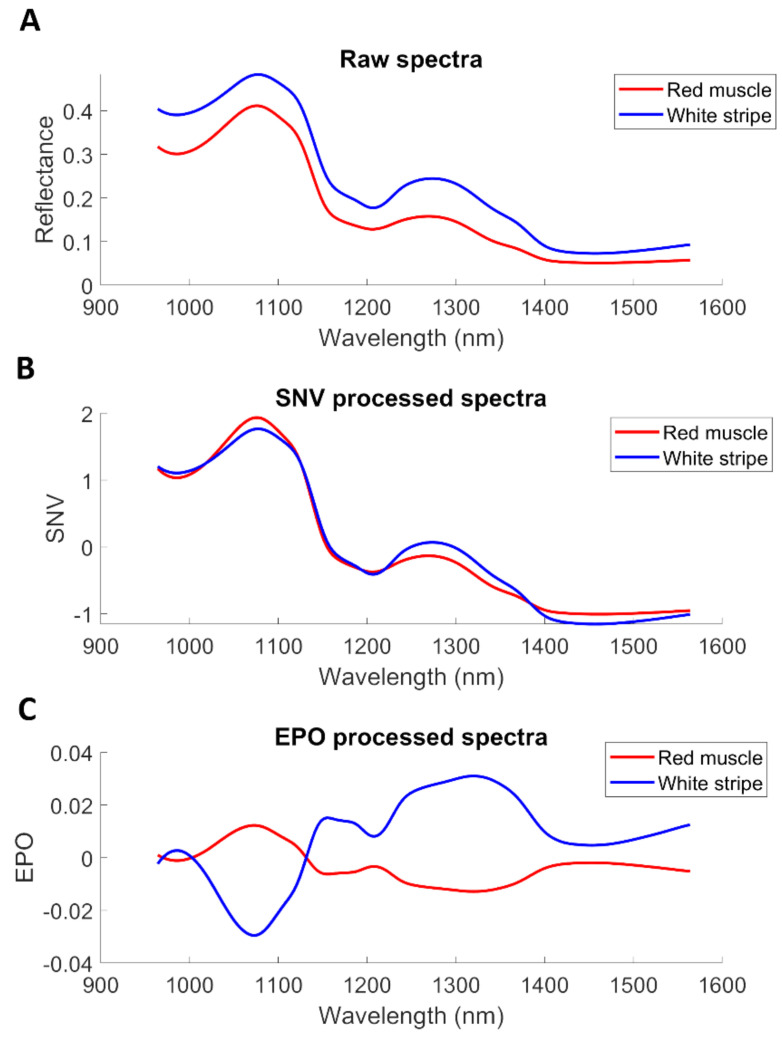
Influence of different spectral pre-processing methods on the mean spectra of one hypercube in the training set. (**A**) Raw spectra; (**B**) Pre-processed spectra with SNV; (**C**) Pre-processed spectra with EPO.

The mean spectra of white stripe and red muscle were computed from the first image of the training set. As seen from Figure 7A, higher reflectance is evidenced in white stripe owing to its bright white appearance. It is also observed that white stripe and red muscle present the different band shapes at 1210 nm and 1450 nm, which can be assigned to second overtone of ${\mathrm{CH}}_{2}$ bond [36] and OH bond [35], respectively. SNV pre-treatment (Figure 7B) is seen to reduce some interfering variability. Using the mean spectrum of each fillet as the interference, the spectral difference between red muscle and white stripe is clear to observe after employing EPO (Figure 7C).

#### 3.2.2. PLSDA and SVM Modelling

The selection of LVs for PLSDA modelling is shown in Figure S7. Table 2 summaries the classification performance of models built for salmon samples. It is noted that the prediction performance varies from sample to sample. For instance, the PLSDA model developed from raw spectra (i.e., PLSDA-I) presented the accuracy of 89.06% for the validation sample, while the inferior predictive ability is witnessed in test sets with the accuracy of 81.02 % for Test 2 fillet. Overall, pre-processing attempts to enhance model performance compared to using raw spectra, which is expected due to the reduction of variance that is irrelevant to classification. SVM outperformed PLSDA under the same pre-treatment condition. The best model performance was found using SVM classifier (SVM-III) built from EPO pre-treated spectra.

**Figure 8:**
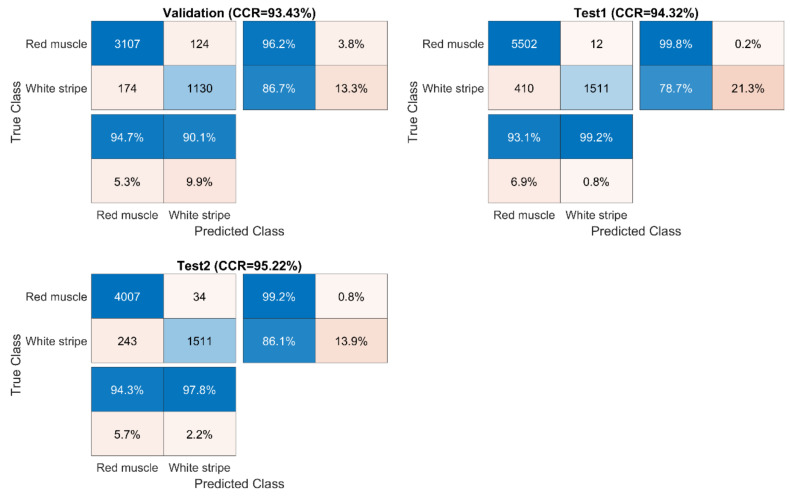
Confusion matrices for validation and test sets obtained by the SVM-III model built from EPO pre-treated spectra.

Confusion matrices for validation and test sets were computed from SVM-III (model built from EPO pre-treated data) due to its better performance in general and displayed in Figure 8. For test sets, it was found that the classification of red muscle pixels has high sensitivity (over 99%), implying the strong ability to correctly identify red muscle pixels on the salmon surface. On the other hand, the sensitivity of identifying white stripe was relatively low, meaning that it is more likely to wrongly classify actual white stripe pixels into the red muscle category.

**Figure 9:**
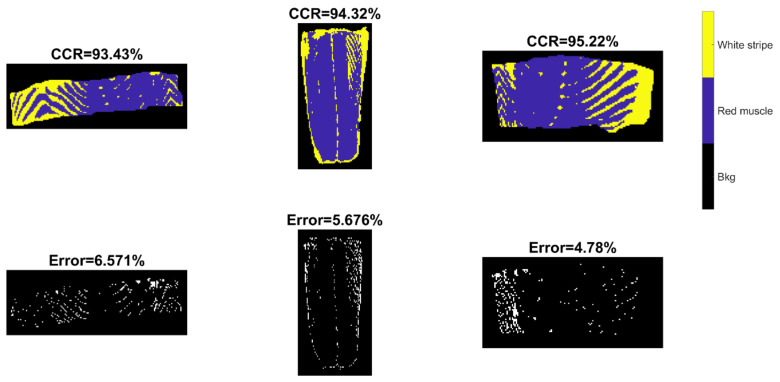
Classification and misclassification maps for validation (the **left** image) and test sets (the **middle** and **right** images) obtained by the support vector machine (SVM) model built from EPO pre-treated spectra (SVM-III of Table 2).

Prediction maps and misclassification maps were subsequently developed from SVM-III and shown in Figure 9. Meanwhile, the ground truth images are displayed in Figure S8. Visually, the misclassified pixels are distributed along the white stripe. It is consistent with the confusion matrix (Figure 8) where the sensitivity of red muscle is much higher than that of white stripe.

#### 3.2.3. PCA-CNN Modelling

**Figure 10:**
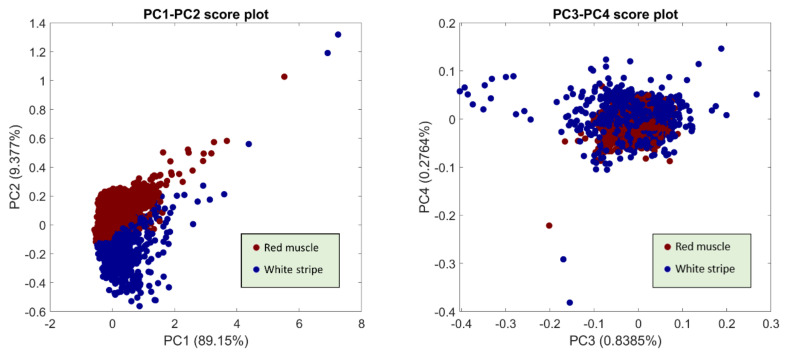
PCA scatter plots from the first four PCs. Explained variance of each PC is indicated in bracket.

PCA is the key to the proposed method which aims to extract spectral and spatial information from hyperspectral images. To visualize PCA results, scores and loadings obtained from EPO pre-processed spectra are presented in serval figures. Figure 10 shows the scatter plots of score values from the first four PCs. It can be seen that PC1 and PC2 express the major difference between these two classes, facilitating the separation into two clusters on the PC1-PC2 scatter plot. In the case of spectral loadings (see Figure S9), it is found that the separation mostly relies on the influence of the band over 1000–1100 nm, 1250–1350 nm, and 1400–1500 nm. Additionally, PC1 and PC2 score images are illustrated in Figures S10 and S11 of Supplementary Materials, respectively. PC1 score images exhibit some noisy pixels corresponding to the pixels with strong signals in the reflectance images of Figure 2. White stripe pixels presenting the blue colors are slightly distinguishable from red muscle pixels in PC2; however, there is an area of red pixels occurred in individual salmon fillet mostly due to the higher thickness. The surface of salmon fillet was usually not flat, with varying thickness from region to region, making it a challenging task for pixel classification.

**Figure 11:**
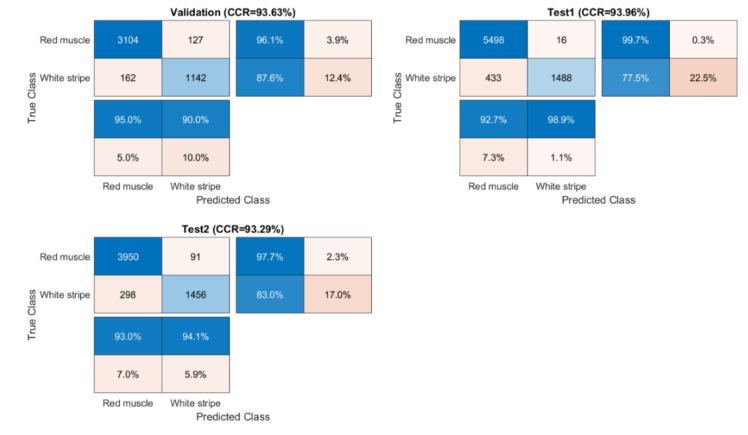
Confusion matrices for validation and test sets obtained by the PCA-CNN model built from EPO pre-treated spectra.

**Figure 12:**
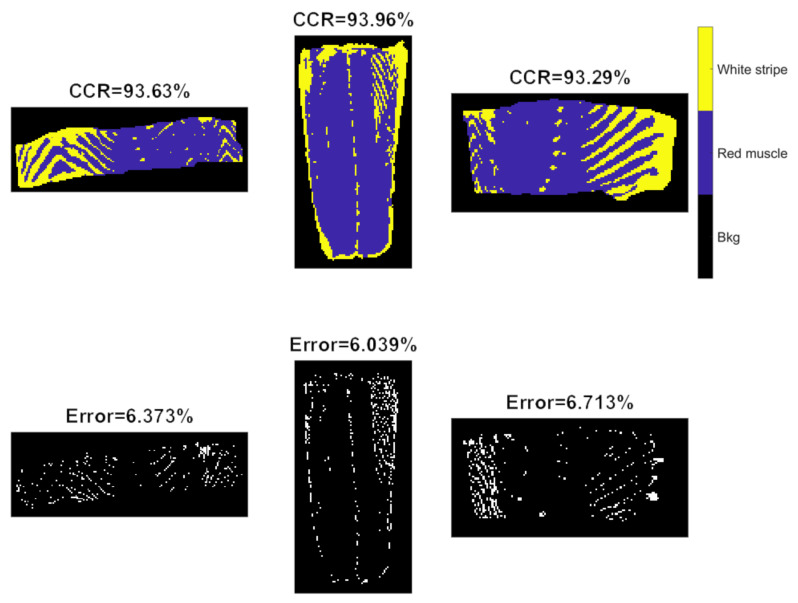
Classification and misclassification maps for validation (the **left** image) and test sets (the **middle** and **right** images) obtained by the PCA-CNN model built from EPO pre-treated spectra.

PCA-CNN models were respectively developed from raw, SNV, and EPO pre-processed spectra, as the results shown in Table 2. In all cases, the window size were set to 7 and the first 5 PCs (explaining 99% of variance for raw spectra, 98% of variance for SNV, and 99% for EPO pre-processed spectra) were used for 2-D CNN modelling, meaning that the classification of pixels was based on the patch of 7×7×5. Figure S12 shows training process for the model built from SNV pre-treated spectra. The accuracy for training and validation both increase and then remain flat, while the loss decreases rapidly at the beginning and keeps stable at the late stage. EPO pre-treatment outperforms SNV with higher accuracy for test sets. EPO pre-treatment improved the accuracy of Test 1 image from 84.76%, using raw spectra to 93.96%. Figure 11 shows confusion matrices for validation and test sets calculated from the PCA-CNN-III (pre-processed with EPO) model. In addition, classification and misclassification maps are exhibited in Figure 12 for visualization purpose.

#### 3.2.4. Three-Dimensional CNN Modelling

**Figure 13:**
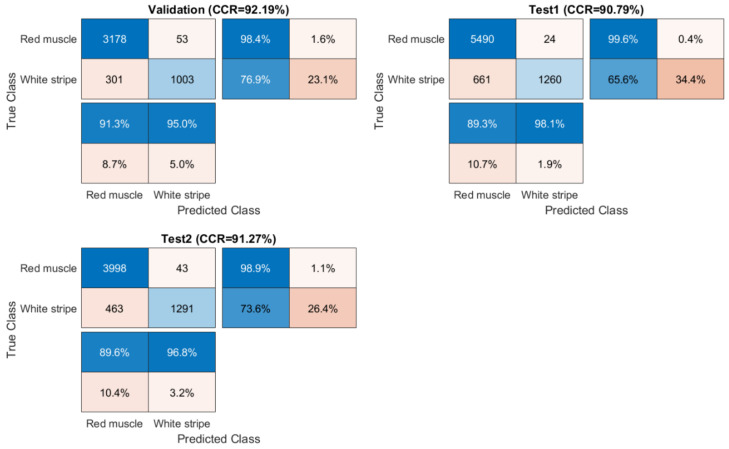
Confusion matrices for validation and test sets obtained by the 3-D CNN model built from EPO pre-treated spectra.

**Figure 14:**
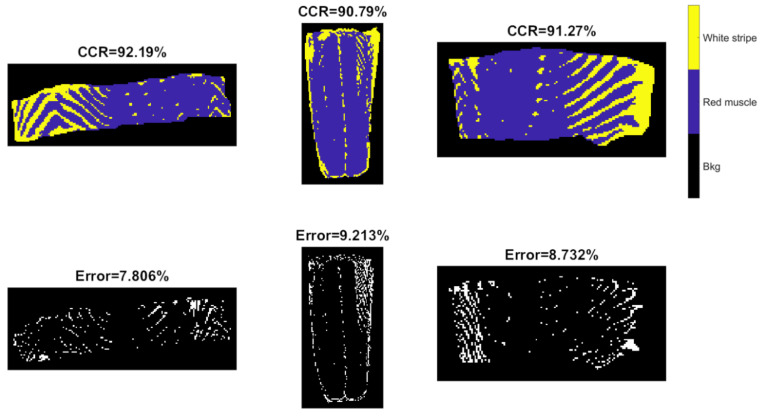
Classification and misclassification maps for validation (the **left** image) and test sets (the **middle** and **right** images) obtained by the 3-D CNN model built from EPO pre-treated spectra.

3-D CNN models were developed from raw, SNV and EPO pre-treated spectra, with the training progress of 3D-CNN-II (SNV pre-processed spectra) shown in Figure S13. Compared to the 2-D CNN model (Figure S12), similar curve shapes (i.e., accuracy and loss) are noticed. Originally, the training set was characterized as 5-dimensional dataset (7×7×180×1×21222 where 7 represents the window size of each patch, 180 denotes to the number of spectral variables, and 21,222 refers to the number of observations). However, CNN training produced a “CPU out of memory” error message. As a result, we extracted every fourth observation and formed a reduced training set with the size of 7×7×180×1×5306. Again, spectral pre-treatments improved the model performance, which was more obvious for the test sets. According to Table 2, the 3D-CNN-III model leads to the accuracy of 92.19%, 90.79%, and 91.27%, respectively, for validation of the Test 1, and Test 2 images, which are inferior to that of the PCA-CNN-III model. Confusion matrices for validation and test sets are computed and illustrated in Figure 13. There is little distinguishable difference in classification maps obtained from PCA-CNN-III (Figure 12) and 3D-CNN-III (Figure 14), although less misclassified pixels are found for PCA-CNN-III on closer inspection.

For classification of white stripe pixels from red muscle, the best performance was achieved by using SVM developed from EPO pre-treated spectra (SVM-III), followed by PCA-CNN-III. This is probably because the spatial information was unable to make much contribution for this classification task. Deep learning strategies (PCA-CNN and 3-D CNN) show better predictive ability than PLSDA with overall higher accuracy. In terms of running time, 3-D CNN is the most time-consuming, while SVM and PLSDA are the fastest. In spite of using a reduced training set equivalent to 25% of original data, 3-D CNN (43 min) is still much slower than 2-D CNN training (7 min) given the same programming environment.

## 4. Discussion

This work intends to compare different supervised classifiers for NIR hyperspectral imaging data acquired from benchtop instruments. Using PLSDA, the relevant sources of data variability are modelled by LVs, which are a linear combination of the original variables, and, consequently, it allows graphical visualization and understanding of the different data patterns and relations by LV scores and loadings. Theoretically, PLSDA combines dimensionality reduction and discriminant analysis into one algorithm and is especially applicable to modelling high dimensional data. Therefore, it has demonstrated great success in modelling hyperspectral imaging datasets for diverse purposes. In this work, however, PLSDA presented the inferior modelling performance in both example datasets.

For sweet samples, there are distinctive spatial patterns among classes, which could potentially contribute to the classification. PLSDA and SVM focus exclusively on the spectral domain despite the inherent spatial-spectral duality of the hyperspectral dataset. In other words, the hyperspectral data are considered not as an image but as an unordered listing of spectral vectors where the spatial coordinates can be shuffled arbitrarily without affecting classification modeling results [37]. As we can observe from classification and misclassification maps of sweet samples, PLSDA and SVM classifiers exhibit random noise in pixel-based classification (significantly less in the CNN-based methods), because they ignore spatial-contextual information when providing a pixel prediction. Therefore, pixel-based CNN models outperform traditional chemometric technique (i.e., PLSDA) and machine learning (SVM) in terms of every aspect of classification performance for sweet samples, e.g., higher accuracy, sensitivity, and precision. On the other hand, the spectral difference is the main source for classification between white strip and red muscle classes of salmon samples. Therefore, inclusion of spatial information by applying CNN based strategies (i.e., PCA-CNN and 3-D CNN) cannot necessarily enhance model performance.

PCA-CNN and 3-D CNN both enable to use the conjunction of spatial and spectral information, therefore achieving better classification results compared to PLSDA. PCA is based on the fact that neighboring bands of hyperspectral images are highly correlated and often convey almost the same information about the object. In this sense, PCA facilitates to transform the original data so to remove the correlation among the bands. Technically, the first few PC score images may contain most of the information contained in the entire hyperspectral image data; hence, classifications using the most significant PCA bands yield the same class patterns as when entire hyperspectral data sets are used. Our two hyperspectral datasets (i.e., sweet and salmon) suggested that similar predictive capability is witnessed between these two CNN-based strategies. However, in terms of the runtime of modelling training, the PCA-CNN classifier requires much less time than 3-D CNN partly due to the computational complexity of the 3-D convolution layer. Moreover, it is observed that the number of parameters that each model needs to adjust during the training phase, being the PLSDA model with fewest parameters and the 3-D CNN the one with the most parameters to fit.

## 5. Conclusions

In this work, PLSDA, SVM, PCA-CNN, and 3-D CNN models for pixel classification were developed and compared in terms of accuracy and efficiency. The results from sweet dataset strongly support the fact that joint spectral and spatial features are more useful than focusing only on spectral features, making the CNN-based modelling ideal for the extraction of highly discriminative features for classification purposes. Nevertheless, salmon dataset demonstrated that SVM model outperformed CNN based methods because spatial information is less important for this classification task. PCA-CNN and 3-D CNN delivered similar classification results, yet the run-time to implement PCA-CNN is much faster than 3-D CNN, suggesting that the use of the PCA approach prior to hyperspectral image classification is beneficial and effective. It significantly reduces the amount of data to be handled and achieves practically acceptable and accurate classification results that are comparable with those obtained using the entire hyperspectral image data. This work also demonstrated the significance of applying spectral pre-processing techniques to complex HSI scenes before classification. Although CNN modelling is powerful in feature extraction, spectral pre-processing techniques manage to remove or reduce some unwanted variance and therefore enhance the classification performance. The proposed CNN based modelling framework from this work could be adopted for solving similar classification problems in food and agriculture applications.

## Figures and Tables

**Table 1:** Model performance of sweet samples for validation and test images in terms of % correct classification rate (%CCR).

Model	Pre-Treatment	Time (min)	Training	Validation	Test1	Test2
PLSDA-I	-	<1	97.02	99.42	99.55	98.37
PLSDA-II	SNV	<1	99.37	99.41	99.58	99.16
PLSDA-III	SG+SNV	<1	99.44	99.33	99.69	99.52
SVM-I	-	<1	99.59	99.61	99.62	99.29
SVM-II	SNV	<1	99.99	99.97	99.83	99.69
SVM-III	SG+SNV	<1	99.99	99.97	99.80	99.70
PCA-CNN-I	-	2	99.95	99.01	99.39	99.00
PCA-CNN-II	SNV	2	100	98.79	100	100
PCA-CNN-III	SG+SNV	2	100	100	100	100
3D-CNN-I	-	14	97.72	98.64	98.05	97.94
3D-CNN-II	SNV	13	100	100	100	100
3D-CNN-III	SG+SNV	13	100	100	100	100

Note: PLSDA: partial least squares discriminant analysis; SVM: support vector machine; PCA: principal component analysis; CNN: convolutional neural network; SNV: standard normal variate; SG: First derivative using Saviztky-Golay.

**Table 2:** Model performance of salmon samples for validation and test images in terms of % correct classification rate (%CCR).

Model	Pre-Treatment	Time (min)	Training	Validation	Test1	Test2
PLSDA-I	-	<1	92.89	89.06	82.54	81.02
PLSDA-II	SNV	<1	92.48	93.10	83.75	87.97
PLSDA-III	EPO	<1	90.37	87.81	89.00	85.85
SVM-I	-	<1	94.13	93.72	83.27	87.37
SVM-II	SNV	<1	94.96	87.74	83.81	80.85
SVM-III	EPO	<1	93.02	93.43	94.32	95.22
PCA-CNN-I	-	8	94.51	92.97	84.76	87.85
PCA-CNN-II	SNV	7	94.46	92.86	84.32	85.76
PCA-CNN-II	EPO	7	95.02	93.63	93.96	93.29
3D-CNN-I	-	43	92.76	92.17	81.99	85.44
3D-CNN-II	SNV	43	96.21	93.16	84.87	87.71
3D-CNN-III	EPO	43	93.25	92.19	90.79	91.27

Note: PLSDA: partial least squares discriminant analysis; SVM: support vector machine; PCA: principal component analysis; CNN: convolutional neural network; SNV: standard normal variate; SG: First derivative using Saviztky-Golay.

## Supplementary Materials

The following are available online at https://www.mdpi.com/1424-8220/20/18/5322/s1, Figure S1: Mean images of the test set with mixed sweet samples at the spectral domain, Figure S2: The evolution of accuracy with the number of LVs developed from (A) raw spectra; (B) SNV pre-treated spectra; (C) first derivative spectra followed by SNV pre-treatment, Figure S3: PC1 score images for the training and validation sets, Figure S4: PC2 score images for the training and validation sets, Figure S5: Training progress of PCA-CNN-III with accuracy (top) and loss (bottom) plotted against iteration, Figure S6: Training progress of 3-D CNN with accuracy (top) and loss (bottom) plotted against iteration, Figure S7: The evolution of accuracy with the number of LVs developed from (A) raw spectra; (B) SNV pre-treated spectra; (C) EPO pre-processed spectra, Figure S8: Ground truth for the validation and test sets, Figure S9: PCA loadings for the first four PCs, Figure S10: PC1 score images of all salmon fillets, Figure S11: PC2 score images of all salmon fillets, Figure S12: Training progress of PCA-CNN-II with accuracy (top) and loss (bottom) plotted against iteration, Figure S13: Training progress of 3D-CNN-II with accuracy (top) and loss (bottom) plotted against iteration.
