# Supplementary material for: Deep Spectral-Spatial Features of Near Infrared Hyperspectral Images for Pixel-Wise Classification of Food Products
Source: Sensors (Basel). 2020 Sep 17;20(18):5322. doi: 10.3390/s20185322 (PMC7570506; doi:10.3390/s20185322)
Supplement: Supplementary file 1 [file sensors-20-05322-s001.pdf]

Deep spectral-spatial features of near infrared hyperspectral images for pixel-wise classification of food products

Hongyan Zhu[1], Hailin Feng[3], Keping Yu[4], Aoife Gowen[2], Jun-Li Xu[2*]

[1]*College of Electronic Engineering, Guangxi Normal University, Guilin 541004, Guangxi, People's Republic of China*

[2]*UCD School of Biosystems and Food Engineering, University College of Dublin (UCD), Belfield, Dublin 4, Ireland*

[3]*School of Information Engineering, Zhejiang A&F University, Hangzhou, China*

[4]*Global Information and Telecommunication Institute, Waseda University, Shinjuku, Tokyo, 169-8050, Japan*

*Corresponding author: E-mail: junli.xu@ucdconnect.ie.

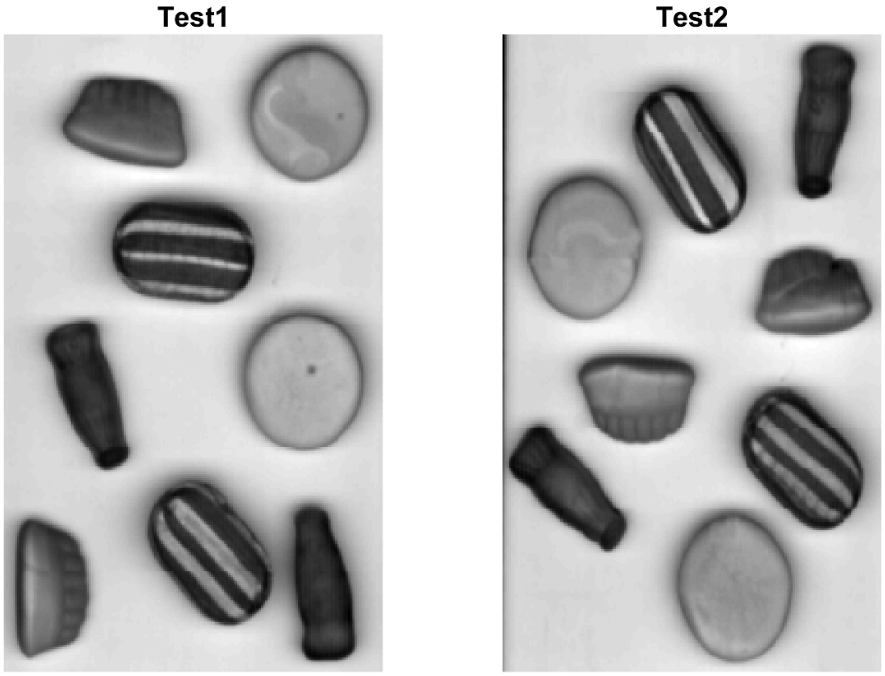

Figure S1. Mean images of the test set with mixed sweet samples at the spectral domain.

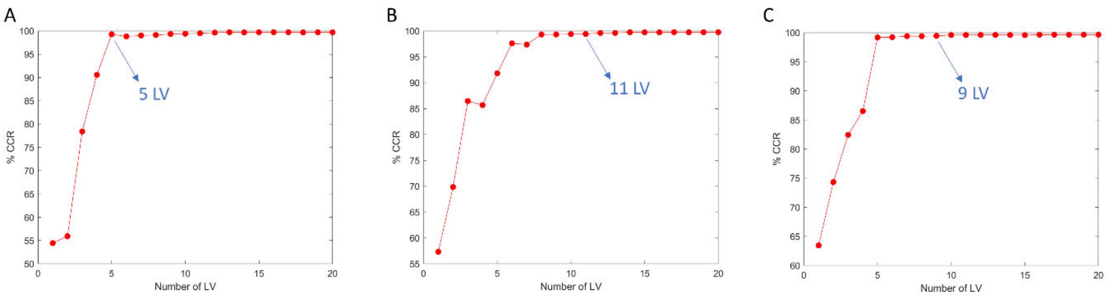

Figure S2. The evolution of accuracy with the number of LVs developed from (A) raw spectra; (B) SNV pre-treated spectra; (C) first derivative spectra followed by SNV pre-treatment.

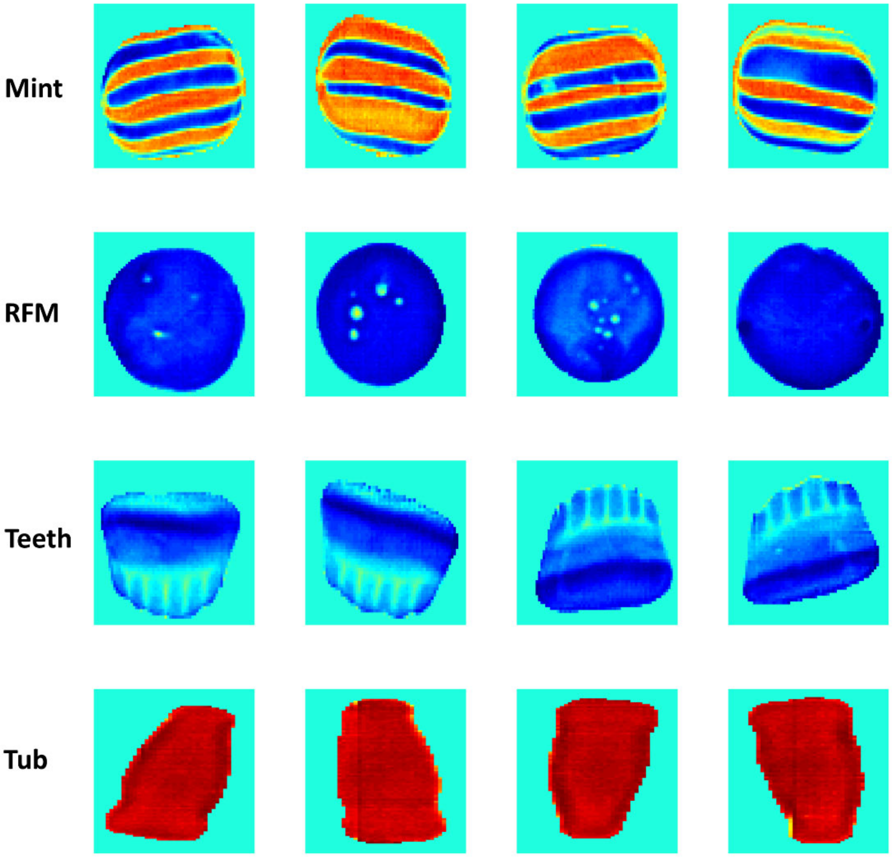

Figure S3. PC1 score images for the training and validation sets.

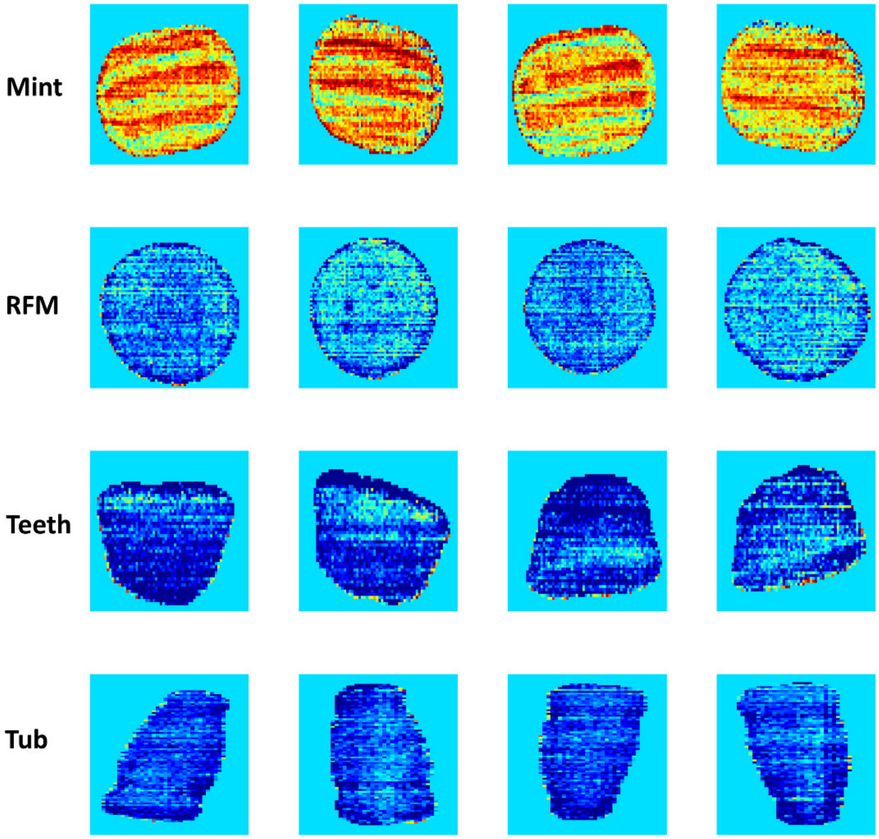

Figure S4. PC2 score images for the training and validation sets.

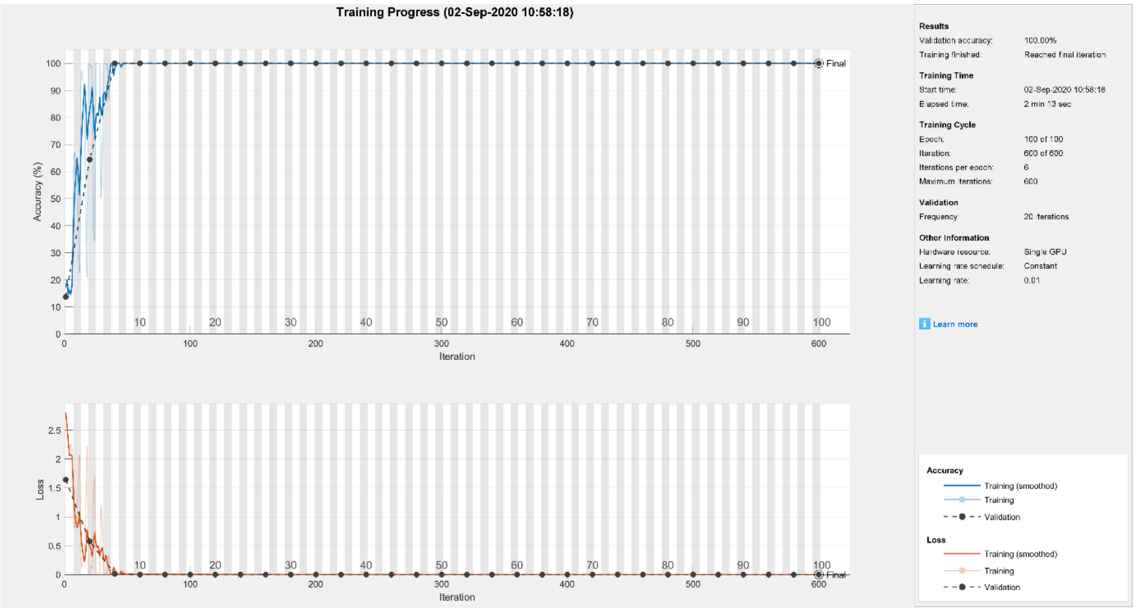

Figure S5. Training progress of PCA-CNN-III with accuracy (top) and loss (bottom) plotted against iteration.

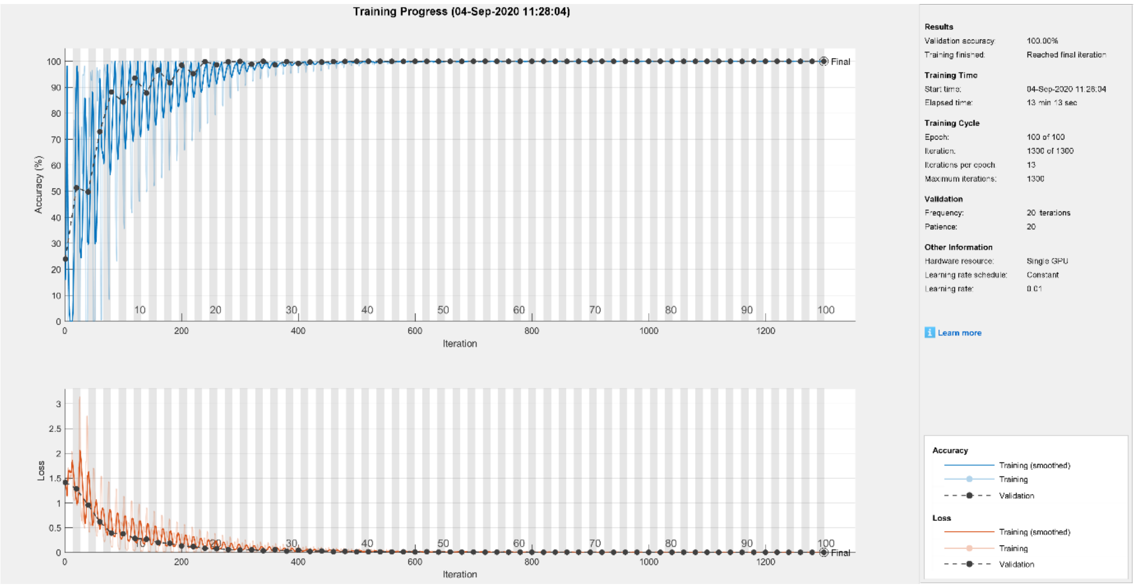

Figure S6. Training progress of 3-D CNN with accuracy (top) and loss (bottom) plotted against iteration.

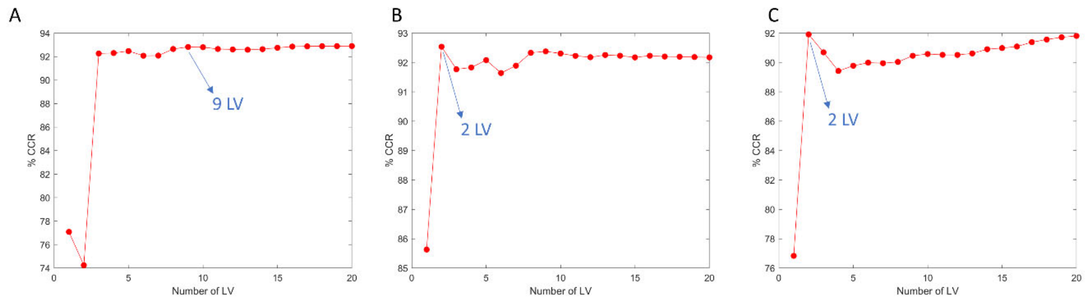

Figure S7. The evolution of accuracy with the number of LVs developed from (A) raw spectra; (B) SNV pre-treated spectra; (C) EPO pre-processed spectra.

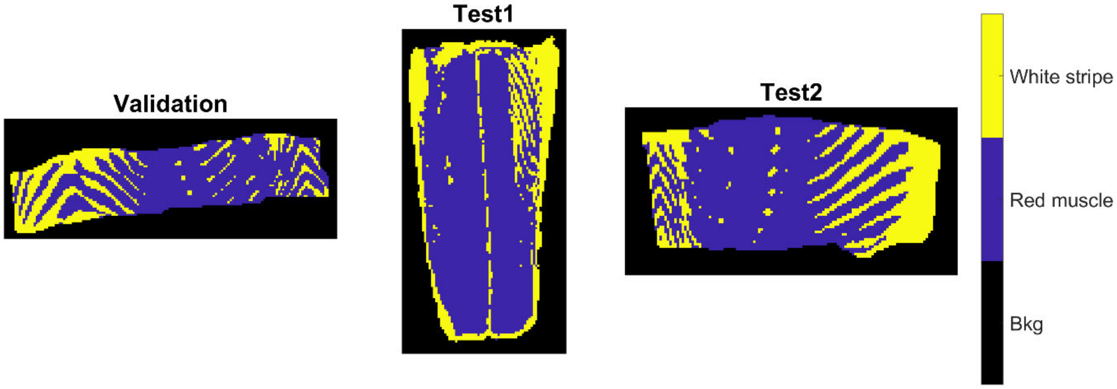

Figure S8. Ground truth for the validation and test sets.

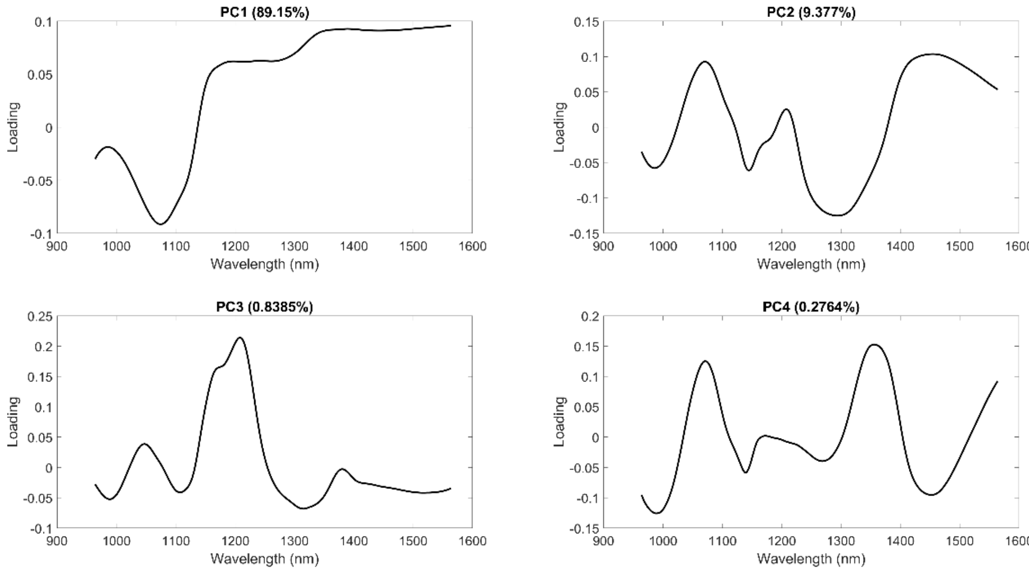

Figure S9. PCA loadings for the first four PCs.

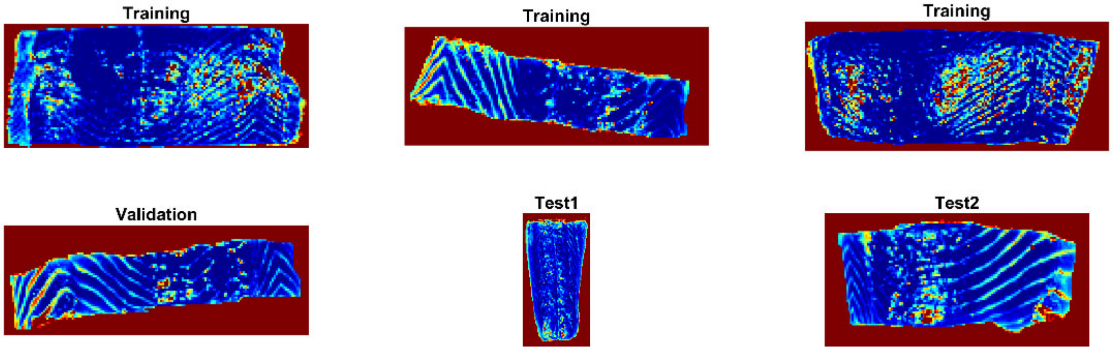

Figure S10. PC1 score images of all salmon fillets.

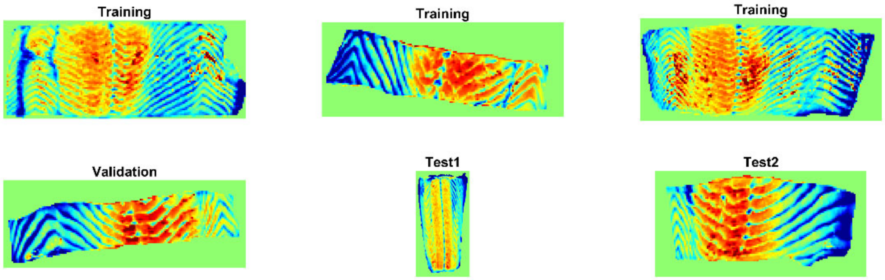

Figure S11. PC2 score images of all salmon fillets.

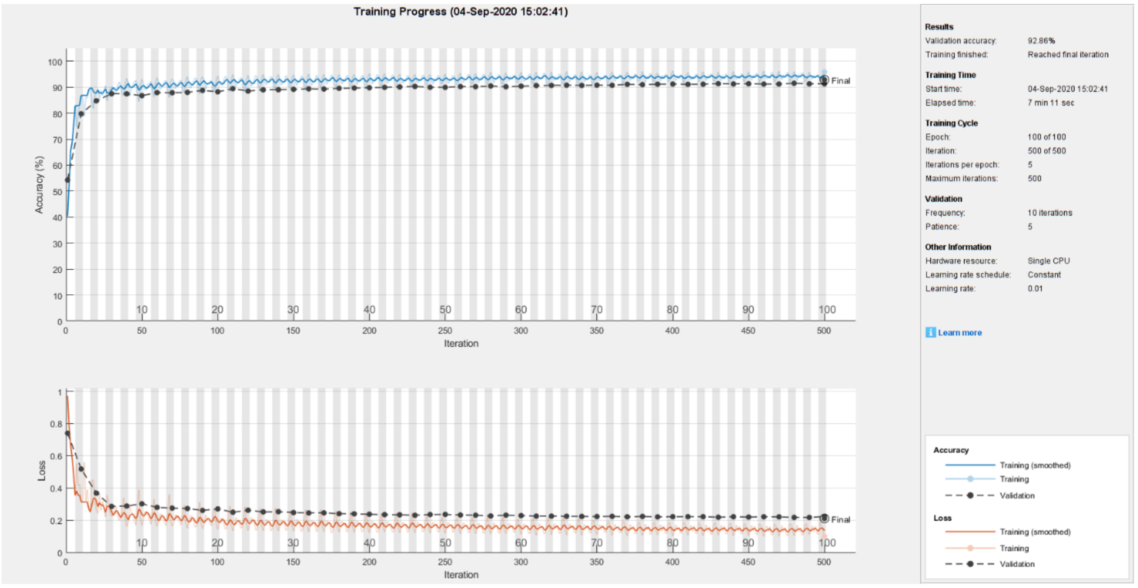

Figure S12. Training progress of PCA-CNN-II with accuracy (top) and loss (bottom) plotted against iteration.

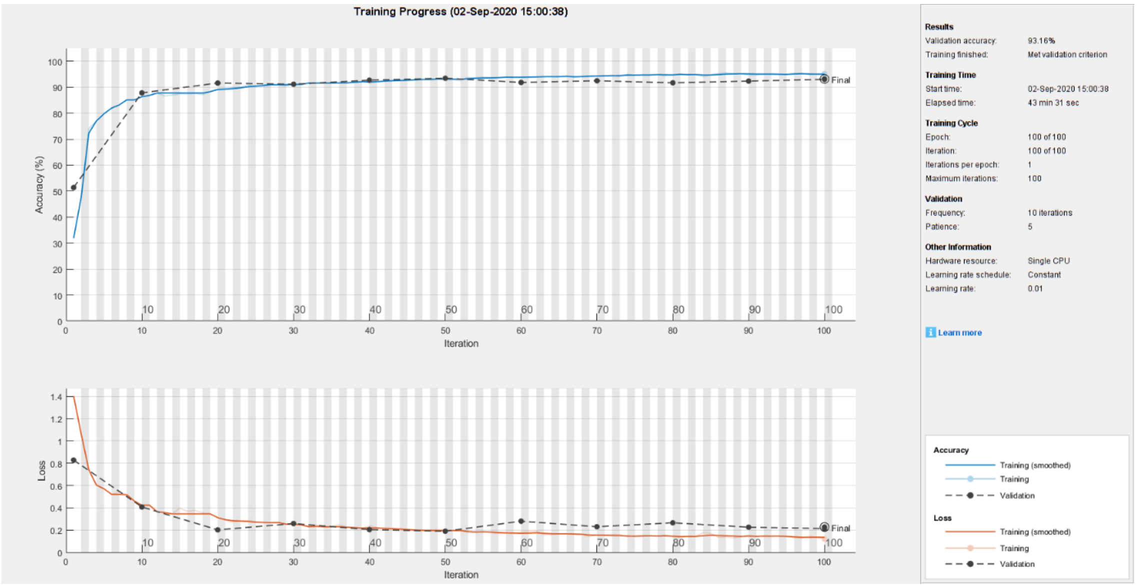

Figure S13. Training progress of 3D-CNN-II with accuracy (top) and loss (bottom) plotted against iteration.
